# Supplementary material for: Mortality, morbidity and clinical care in a referral neonatal intensive care unit in Haiti
Source: PLoS One. 2020 Oct 14;15(10):e0240465. doi: 10.1371/journal.pone.0240465 (PMC7556516; doi:10.1371/journal.pone.0240465)
Supplement: Attachment — Submitted filename: Response to Reviewers.doc [file pone.0240465.s001.doc]

Beatrice E. Lechner MD

Department of Pediatrics

Phone 401-274-1122 ext 47438

Fax: 401-453-7571

Email: [blechner@wihri.org](mailto:blechner@wihri.org)

Dr. Joerg Heber

Editor in Chief

PLOS One

September 9, 2020

Dear Dr. Heber,

We are pleased that you have allowed us to resubmit a revised version of our manuscript “Mortality, morbidity and clinical care in a referral neonatal intensive care unit in Haiti” to *PLOS One* for consideration for publication.

We thank the reviewers for their encouraging comments and their thorough and helpful critique. We have carefully considered their comments and have made changes based on them that we believe improve the overall manuscript. Most noteworthy is the addition of further details in Methods as well as the addition of study limitations to the Discussion. Please find below a point-by-point response to the Reviewer’s critique.

**Reviewer 1**

Critique 1: “*The method section only contains a few words on the statistical analyses performed. While they provided enough information on the study site in the background section, more information should be provided on the following elements: a. The study design; b. inclusion and exclusion criteria; c. the outcome definition and calculation; need to be defined; d. and data quality (Given the inherent limitation associated with data extracted from existing medical records).”*

We have added additional information on the statistical analysis to the methods section (lines 130-135), as well as further clarifications on points a and b (lines 110, 116-117, 125-126). We added a paragraph on point d to the Discussion (lines 226-279). Given that this study is a descriptive study, an outcome definition or calculation is not appropriate.

Critique 2: *“The way the results were being interpreted suggest an analytic study that would aim to establish the risk factors to neonatal death at the study site, which would require other types of study designs and statistical analysis. For instance, the following sentence clearly suggests that a relative risk has been estimated to establish the association between the risk factors and the outcomes: "The diagnoses associated with an increased risk of death included prematurity, RDS, sepsis, and kernicterus. Diagnoses associated with a decreased risk of death were chorioamnionitis, transient tachypnea of the newborn (TTN), and jaundice (Table 1)". Another example is this sentence: "Intubation was associated 158 with death (total intubations n=8; intubation among infants who died n=6; 159 p=0.003)". The most important point here is the need to keep the result interpretation consistent with the study objectives and methods.”*

In order to clarify the objectives of the study and thus the interpretation of the results more clearly, we have edited the abstract and background (lines 32-34, 102-104) to state that this study seeks to describe clinical care in the St. Damien NICU in order to target limited resources toward improving outcomes. We did not calculate relative risks. In order to avoid confusion, we changed the word “risk” to “outcome” to more accurately reflect our analysis (lines 146-147, 209). Finally, this retrospective cohort study is adequate to describe the relationship of characteristics with death, without positing that these are causes.

Critique 3: *“The study limitations that are subject to affect both internal and external validity should be clearly stated and particularly those inherent to the study design itself.”*

We have added a discussion of internal and external validity to the Discussion (lines 266-279).

Critique 4: *“The conclusion seems to suggest that these findings could be extrapolated to the whole country should be reconsidered once the study limitations are being clearly stated.”*

We have clarified in the Discussion (lines 266-279) that there are limitations to the study that preclude extrapolating to all of Haiti based on this study.

Critique 5: *“The authors need to make sure that the most recent pieces of evidence are being considered. For instance, the current neonatal mortality rate in Haiti is 32 per 100 live birth (*[*https://www.dhsprogram.com/pubs/pdf/FR326/FR326.pdf*](https://protect-us.mimecast.com/s/S6nTC82oNWtnPPzJFRbFvV)*) instead of 25 per 100 live birth, as mentioned.”*

Thank you for bringing the more current citation to our attention. We have updated the manuscript (line 60).

Critique 6: *“Need an in-text citation for this sentence: "The large percentage of women who deliver their infants without a skilled healthcare worker present and the significant shortage of neonatal intensive care units contributes to the high neonatal mortality rates"; If available, it would be great if more socio-demographic information (maybe characteristics of the mothers) can be provided in table one beyond age.”*

We have added a citation for the sentence above (line 63). Unfortunately, additional demographic information regarding the mothers beyond what was presented in Table 1 was not available in the charts.

Critique 7: *“The percentage for the other diagnosis needs to be provided, including the n value "The most common diagnosis was sepsis, at 91.5%. Other common diagnoses 149 were chorioamnionitis, respiratory distress syndrome, jaundice, prematurity, and perinatal asphyxia (Table 1).”*

The percentages, as well as the n values, for each diagnosis, are listed in Table 1.

Critique 8: *“It would be great if the author could present table 2 according to the neonates' outcomes as well.”*

We weighed this suggestion in depth. While Table 1 was structured according to the outcomes of death vs. survival so as to demonstrate which pre-existing patient characteristics were associated with death, the role of Table 2 was to show which interventions were available in the NICU and utilized. Thus, demonstrating outcomes according to interventions utilized would not result in meaningful metrics.

Critique 9: *“The authors should describe how data quality may have impacted the outcome (neonatal death) measurement (Potential information bias).”*

We have included this information in the Methods section (line 125).

Critique 10: *“The authors should explore other factors that can affect the Length of Stay in NICU.”*

We have included an exploration of this topic under Discussion (lines 212-215).

Critique 11: “It seems like the authors have enough variable to estimate a GLM to more highlight the roles of the contributors discussed in the neonatal death rate at the study site while clearly state the study limitations.”

While there may be enough deaths in the data to do a GLM (generalized linear model/logistic regression), those models usually involve designating a particular variable as a focal cause of death and include other variables as possible confounders. This is a descriptive study looking at associations of a number of different outcomes relating to death, not one particular thing. Thus, a GLM would not add useful information.

**Reviewer 2**

Critique 1: *“However, despite mentioning the issue of low Skilled Birth Attendants and low number of pediatricians, the authors did not discuss the role of neonatal nurses in such a health care setting as they would normally spend more time in the clinical setting.”*

Thank you for pointing out this oversight on our part. In addition to birth attendants and pediatricians, neonatal nurses play a central role in providing care to neonates. We have added this information to the Introduction (lines 98-100).

We would like to thank the Reviewers again. We hope you will find the revised manuscript acceptable for publication in *PLOS One.*

Sincerely,

Beatrice E. Lechner, MD
